# Exploring the Concepts and Practices of Health and Illness Among the Wixaritari Indigenous People of Western Mexico: An Ethnographic Study

**DOI:** 10.7759/cureus.115238

**Published:** 2026-08-26

**Authors:** Igor M Ramos Herrera, Hilda B Orozco, René C Crocker, Quetzabel C Castañeda, Luciano Sanchez, Leela McKinnon, Eric Shattuck, Thankam Sunil, José L Vázquez, Sandra M Rubio

**Affiliations:** 1 Public Health, University of Guadalajara, Guadalajara, MEX; 2 Social Sciences, University of Guadalajara, Guadalajara, MEX; 3 Anthropology, University of Toronto, Toronto, CAN; 4 Anthropology, Florida State University, Tallahassee, USA; 5 Public Health, University of Tennessee, Knoxville, USA

**Keywords:** culture, health, illness, indigenous peoples, traditional medicine

## Abstract

Introduction

Despite growing research on Indigenous traditional medicine, the literature still lacks studies that examine holistic understandings of health among Indigenous people. This study aimed to explore Wixárika conceptions of health and illness in western Mexico in order to deepen understanding of their health-related practices and guide intercultural strategies to improve their quality of life.

Methods

A qualitative study with an ethnographic approach was conducted among 127 adult Wixárika participants in two contrasting contexts in western Mexico, using semi-structured in-depth interviews and thematic analysis.

Results

Findings show that, for the study participants, health is understood as a state of balance encompassing physical, spiritual, mental-emotional, and economic-occupational dimensions. Illness is perceived as a disruption of this balance, shaping therapeutic practices that combine ancestral knowledge with biomedical care. Functional capacity to carry out everyday activities and economic well-being also emerged as important indicators of health status. The use of traditional remedies alongside Western medicine reflects an adaptive strategy.

Conclusions

This study contributes to a broader intercultural understanding of health and provides a basis for respectful dialogue aimed at promoting the well-being of these Indigenous people.

## Introduction

Concepts of health and illness vary according to the cultural, historical, geographical, and social contexts in which people live. In the Americas, Indigenous people have developed their own perspectives on these matters based on their cosmology and cultural traits [1,2]. Unlike the Western biomedical vision focused on a pathogenic model of health, many Indigenous communities understand health as a balance between the individual, their community, their spirituality, and nature.

In the context of the Indigenous people of the Americas, a diversity of perspectives on health and illness is observed, transmitted across generations through traditional practices, herbal or naturopathic medicine, and rituals and ancestral knowledge [3]. In Mexico, particularly in Jalisco, Indigenous people such as the Wixárika have maintained their own conceptions of health and illness, despite the influence of national and state health systems and policies aimed at their integration [2].

The Wixárika people are one of the most representative Indigenous groups in Mexico, having inhabited the Sierra Madre Occidental for centuries, primarily in the states of Jalisco and Nayarit, and to a lesser extent in Durango and Zacatecas. They are characterized by their strong cultural identity, distinct religious practices, and a cosmology based on a close bond with nature, forged through sustained resistance to colonization by remaining in the mountains and canyons of the Sierra Madre Occidental [4]. The Wixaritari believe that everything in nature is interconnected-the sun, the earth, the water, the wind, the fire, the animals, the plants, and human beings are all part of the same universe. Their gods are related to natural elements, such as Tatewarí (Grandfather Fire), Nakawé (Mother Water), Yurienaka (Mother Earth), and Tayeupa (Father Sun), and they worship their gods through ceremonies, pilgrimages, and offerings. In terms of health, the Wixaritari combine the use of traditional medicine with knowledge transmitted by the mara'akame, shamanic figures responsible for spiritual and physical healing. These practices include the use of medicinal plants, songs, and purification rituals, reinforcing their holistic vision of well-being [2,3].

The scarce information and understanding of Indigenous perspectives on health led a group of researchers to investigate this topic, given the health problems they currently face, such as obesity, diabetes, and tuberculosis-issues that have not been prevented or controlled despite the efforts of the official health system. Therefore, it is considered crucial to explore more deeply how the Wixárika people in Jalisco perceive health, how they try to preserve it, and which elements they consider essential for their well-being-fundamental aspects of their cosmology.

In studying the health of Indigenous people, it is necessary to consider how current migration processes transform and reconfigure their traditional conceptions of well-being, health, illness, and care. The displacement of Indigenous groups from their communities exposes them to contexts where social inequalities, structural racism, and migration policies operate, directly affecting their health and limiting access to culturally appropriate services. These displacement situations generate new intercultural spaces where the health knowledge of these population groups dialogues, negotiates, or comes into conflict with the hegemonic biomedical model. Analyzing this problem requires recognizing the epistemological diversity of Indigenous people, as well as the historical structures that condition their conception of health [5].

This study sought to explore and describe the conceptions and practices of health and illness among the Wixárika Indigenous people in two contrasting contexts in western Mexico: urban migrants residing in the Guadalajara Metropolitan Area (GMA) and members of the rural community of Mesa del Tirador, municipality of Bolaños, Jalisco, through a qualitative study with an ethnographic approach that used in-depth interviews and participant observation.

Conceptions of health and illness among Indigenous people in Mexico and Latin America

Previous studies suggest that, although there is research on traditional medicine and intercultural health systems, there is still a gap regarding the very perception of the concept of health among Indigenous people [6]. Some research on these people has documented the use of medicinal plants and the importance of shamans or healers in healthcare [7,8], but few have addressed how these communities conceptualize health in holistic terms.

The conceptions of health and illness of Indigenous people in Mexico and Latin America fall within a complex but coherent system of Indigenous traditional medicine, which constitutes a body of knowledge that interprets the relationships of human groups in their daily, social, religious, and health activities. This relationship has been approached from the field of intercultural health, which emerged in the 1950s to describe the relationship between state policies and Indigenous cultures; today, it seeks to integrate Indigenous knowledge about the body and health into the Western model. This approach advocates for the recognition of the intracultural cosmologies and worldviews of these people. These worldviews support an integral vision of health often expressed through concepts such as "Buen Vivir" or "Sumak Kawsay" [9].

The Indigenous health model, according to Aguilar-Peña and colleagues [1], is based on a system of knowledge, skills, and practices rooted in ancestral beliefs and experiences, seeking to preserve health and treat physical and mental illnesses. This holistic approach considers not only individual habits but also harmony with nature, the spirit, the gods, and the community.

For Aguirre-Acevedo and colleagues [10], in Indigenous communities, health is an integral process interconnected with its sociocultural, organizational, religious, and environmental dimensions. Their conception of health and illness is closely linked to Mother Earth and its environmental conditions-aspects of their worldview that directly influence their well-being. For Colombian Indigenous groups like the Emberá, this link is associated with a magical perspective, while the Zenú relate it to spiritual harmony and daily life, which allows for maintaining community and individual balance. Within this worldview, figures like the Jaibaná, a sage and healer, play a key role in connecting nature with health, reaffirming the interdependence between spiritual and physical well-being.

In Mapuche culture in Chile, as described by Alarcón Muñoz and colleagues [6], health and well-being (küme mongen and küme felen) represent harmony with nature and the community. This balance is reflected in healthy growth, food, social development, and the expression of affection in childhood. Furthermore, health implies connection with the itrofill mongen, balance with natural elements, and is associated with values such as respect and generosity, essential for individual and collective well-being.

Carretero Salazar [11], in their research with machi healers of the Mapuche people in Chile, defines health as a connection of spiritual beliefs and energy interactions. Meanwhile, Cedrón Tello [12] describes that for the Yánesha in Peru, health integrates the physical, relational, and spiritual dimensions. A strong body allows for balanced work, while relational well-being depends on harmony with nature. Spiritual health is linked to the union of soul and body and the use of medicinal plants, combining cosmology and Christian faith. Being healthy implies being joyful, active, and engaging in productive activities.

The Ministry of Health and Social Protection [13] mentions that, in some people, the notion of buen vivir (good living) is incorporated, which goes beyond individual health and encompasses harmonious interaction with the universe, spirituality, Mother Nature, and the balance between territory and communities. Palacios Salazar [14] mentions that good health requires harmonious relationships between human-nature, human-territory, human-community, government, authority, and organization. For its part, Crocker Sagástume et al. [3] describe that for the Wixárika people, health is an integral balance connecting the biological, spiritual, social, and natural. Their diet, more than a physical necessity, has a sacred meaning, with rituals that honor the gods and reinforce their cultural identity. Corn is the axis of their cosmology, accompanied by beans and squash, as well as wild foods that strengthen their bond with nature. Harmony with the environment and the community is essential for their well-being. In this same sense, the concept of health for the Wixárika people, according to Verdín Amaro and Santos García [15], is based on their worldview, where well-being implies a balance between the individual, their community, their deities, and their environment.

## Materials and methods

Between 2022 and 2024, the research team from the International Network of Studies on Indigenous Health in Jalisco, which integrates researchers from the University of Guadalajara, Florida State University, the University of Toronto, and the University of Tennessee, Knoxville, conducted a mixed-methods study aimed at understanding sleep and wake habits among Indigenous people living in the northern region of the state of Jalisco and the GMA. This study sought to explore and describe the conceptions and practices of health and illness among Wixárika Indigenous people in two field sites in western Mexico: communities in the Sierra and the GMA, through a qualitative study with an ethnographic approach that used in-depth interviews and participant observation.

This research was developed from an epistemological perspective based on analogical hermeneutics, which allowed interpreting the knowledge, practices, and conceptions of health of Indigenous people without reducing them to a single meaning or losing the richness of their cultural diversity. This approach provided a balanced framework to understand the meaning each community attributes to health and illness, recognizing both common elements and differences specific to each context, promoting a respectful dialogue between Indigenous and academic knowledge [16].

Adult members of the Wixárika ethnic group were invited to participate in this work during three study periods: June 2022, February 2023, and March 2024. During the first two periods, we visited some communities residing in the GMA, which is known to be home to Indigenous people who have migrated from their villages in northern Jalisco to work in the city. In 2020, the GMA had just over 5.2 million inhabitants and is home to approximately five thousand Indigenous migrants; the Wixaritari cluster in the Nuevo México, Valle de los Molinos, and Albaterra neighborhoods in the municipality of Zapopan, and in some neighborhoods of the municipality of Tlaquepaque, but are more dispersed. During the third study period, a visit was made to the community of Mesa del Tirador, municipality of Bolaños, Jalisco, where approximately two thousand people live. This community is one of the closest to the GMA and very near the state of Nayarit, from which they obtain goods and some communication services.

Participants from these localities were included in the study with the help of Wixárika research assistants and through snowball sampling. All were over 18 years of age and openly declared belonging to this ethnic group.

Procedures

Study participants provided informed consent (UTSA IRB #20-023), and all procedures were carried out in Spanish. When necessary, translation from Spanish to Wixárika was performed by the research assistants. All interview questions were translated into the Wixárika language by a native speaker. Thematic analysis was used to identify central themes and interpret the meaning of health and illness in participants' narratives.

Participant Recruitment and Sampling

Initial access to the Wixárika community was negotiated directly with community authorities, who informed community members at a community meeting that the research team would be visiting. During the visit, individuals present in their homes-who had been previously notified-were invited to participate. Snowball sampling was subsequently used to identify additional participants. All participants were selected based on predefined inclusion criteria.

Data Collection

A single semi-structured interview was conducted with each of the 127 adult participants, lasting between one and one and a half hours. Interviews were guided by a topic guide covering the domains reported in this article and were conducted at participants’ homes. Two researchers were present at each interview: the first researcher led the interview while the second observed the process. A community assistant from the Wixárika community served as interpreter when participants did not speak Spanish. Interviews were audio-recorded with participants’ responses in Spanish and subsequently transcribed verbatim.

Thematic Analysis

Prior to data collection, a preliminary set of analytical categories was developed based on the interview guide domains. Following data collection, interviews were printed and coded using these categories. A codebook was developed incorporating the following analytical categories: concepts of health and illness, care practices, and the four dimensions of health (physical, spiritual, mental-emotional, and economic-occupational). The codebook underwent adjustments during the coding process. Three researchers participated in the coding process; disagreements were resolved through group discussion. Data saturation was assessed to determine sample adequacy and was confirmed. Results were differentiated by group, and where differences existed, these were noted in the document. The four analytical dimensions (physical, spiritual, mental-emotional, and economic-occupational) were derived through an iterative process combining inductive coding of participants’ narratives with existing theoretical frameworks on Indigenous health. While the initial codebook was informed by prior conceptual categories, these were refined and validated against the data throughout the analysis, ensuring that the final dimensions reflect the participants’ own expressed conceptions of health and illness.

Reflexivity and Member Checking

The research team included both external academic researchers and two authors who are themselves members of the Wixárika community, which strengthened the cultural grounding of the study. Additionally, one senior researcher (R.C.S.) has maintained an ongoing collaborative relationship with Wixárika communities for over 40 years. Wixárika research assistants participated not only in data collection as bilingual interpreters but also in the verification of findings and the final review of the manuscript, ensuring that the results accurately reflect the community’s own conceptions and practices. The team engaged in ongoing critical reflection on positionality and the potential influence of both insider and outsider perspectives on data interpretation.

Relationship to the Sleep Study

The recruitment and data collection procedures for this study were shared with a concurrent study on sleep and physical activity among the same population. During each interview session, both a sleep and health questionnaire and the semi-structured interview for the present study were administered independently. The thematic analysis reported here pertains exclusively to the qualitative interview data.

This study was conducted in accordance with the provisions of the Mexican General Health Law regarding research and was approved by the research and ethics committees of the Regional Institute for Public Health Research of the University of Guadalajara, under registration number PRO-12/16. It was also registered and approved by the research and ethics committees of the University Center for Health Sciences of the University of Guadalajara under number 20-49.

Gatekeeper negotiation with the communities was conducted through prior meetings with traditional authorities to request access to the community and permission to carry out the study. The authorities were informed of the project and authorized interviews with members of their respective communities. Home visits and visits to their workplaces (craft markets) were made, the reasons for the study were explained to participants, and their consent was requested.

## Results

Sociodemographic description

The 127 participants were adults aged 18 to 65 years; at the time of the study, 61 lived in the GMA, while the remaining 66 lived in the rural community of the municipality of Bolaños. Rural residents were significantly older than their counterparts in Guadalajara (p ≤ 0.001), with an overall mean age of 33.6 years (range: 18-65). Women constituted 68.5% of the participants. Rural residents reported worse physical and mental health but a greater connection to nature.

Concepts of health and illness

The perceptions of the Wixárika participants regarding health and illness reflect a diverse understanding, shaped by their personal experiences, beliefs, and sociocultural contexts. In general, our participants consider maintaining balance as a fundamental point of their well-being, as they themselves express:

"I think that, as a whole, it generates a balance in which you will say or have the capacity to say, well, I'm fine, right? To the extent that you have these elements balanced..." (C3).

Thus, an integral balance must exist between physical, spiritual, mental-emotional, and economic-occupational aspects, allowing them to carry out their daily household activities, work, sleep, and eat, among others, and therefore maintain their health. These aspects are explained below.

Physical aspects

The physical aspect is fundamental for the balance of health among the Wixaritari, as it reflects the harmony between their body and the environment. To maintain their physical well-being, they recognize the importance of adequate nutrition, sufficient water consumption, physical activity, working, rest periods, and adopting preventive measures against illnesses, as explained below.

Most participants expressed that maintaining good physical health is closely related to having an adequate diet, which means eating enough and at regular times, even if only simple foods like beans, and staying hydrated with sufficient water, as one participant comments in their own words: "...I've always tried to eat healthy, even if it's just beans, whatever there is, try to eat healthy; for me, health is very important because it also makes you last longer..." (A3); another participant confirms, "...at least I try to, to drink water. I try to drink my two liters daily" (B4).

Participants recognized that consuming fruits, vegetables, and legumes is also a healthy practice, "...I think fruit, vegetables, water. That's the healthiest" (A2). They also greatly value consuming foods they produce or gather from the land themselves, such as corn, beans, squash, nopal, purslane, quelites, sweet potato, and guaje, as another participant expresses: "...we try to eat all natural foods, consume good food, produce your own food, watch your own food grow, everything produced in the field is consumed" (C1). Corn was particularly important for the participants, as it is not only considered a healthy and sacred food but also establishes a direct relationship with Mother Earth, respect for nature, and the fulfillment of traditions; therefore, it is considered an important food for maintaining their health.

Many other frequently consumed products, such as carbonated beverages, bread, and industrialized foods, are considered unhealthy by the Wixaritari, although they mention that consuming these products is a habit that is becoming increasingly frequent among people living in the city, as one explains:

"...in isolated areas, they are the healthiest, because they eat better, have a better diet than we do here [in the city], because, let's say, here we have everything at hand, from soda and everything, and there it's very difficult to get that every day" (C2).

Furthermore, among Indigenous people, staying in motion is present in their daily activities, such as walking long distances, working the land, or gathering food, which promotes an active and resilient body. Most of the people we interviewed agree that you need to move, walk, or work to maintain health: "...they walk all the time, go to their ranches (plots), plant, which is heavy work, or have to walk from one place to another ranch, and there their health is better because they are active all the time..." (A12). For others, physical activity that benefits their health is related to doing or playing a sport. One of them tells us: "...More than anything, whenever I want to feel better, I always prefer to go for a run, something more strenuous. More than anything, sometimes play, go play" (C3). Working or exercising allows them to burn calories and also generates fatigue, which results in better rest when sleeping at night.

In many Indigenous communities, rest is valued as a natural need that must be respected to maintain well-being, as a tired body is more vulnerable to illness. In this sense, some participants considered sleep and rest as important elements for maintaining physical health; if they fail to sleep the necessary hours at night, they believe they will not have enough energy to perform their work or activities effectively during the day. One participant mentions: "...that you sleep well, eat well, sleep well. So that when you wake up, I think you don't\... you don't feel any pain..." (C33). Others mentioned that resting during the day is also a habit that allows them to stay healthy: "...health or being healthy implies other areas, right? The issue of rest..." (C31).

Participants stated that physical health is not limited solely to the absence of disease but implies the adoption of daily habits oriented toward body care and prevention. Within this conception, early attention to signs of discomfort stands out. Participants mentioned that among their habits for maintaining physical health are preventive measures against illnesses, as another participant mentions: "...well, when you have the flu, not going out in the sun, not bathing, not getting wet..." (E2), or another participant tells us: "...with precaution, with face masks and all. Since I attend to people, right? They come, and I have to take care of that, right? So as not to infect other people" (D21).

Although hygiene can be a fundamental aspect of healthcare, it is interesting that only one participant mentioned it as a relevant element: "...For me, being healthy is being healthy, it's taking care of myself, from hygiene onwards, right? Look at my nails, speaking of hygiene, right? Bathing" (D24). This observation suggests that, within their perceptions, other factors have greater weight in constructing what it means to be healthy. On the other hand, not washing hands before eating is a negative practice seen in children, mainly due to parental neglect, as another participant comments, "...the mother is very careless, they don't wash their hands, they start eating while playing..." (A3). By following these principles, participants not only preserve their physical health but also promote their emotional and spiritual stability.

Spiritual aspects

From the cosmology of Indigenous people, physical health cannot be separated from spiritual health, as both aspects are deeply interrelated. Eating consciously, sleeping adequately, keeping the body active, and maintaining hygiene are not only self-care practices but also acts that reflect respect for the body as the dwelling place of the spirit. These daily actions help preserve internal and external harmony, and when performed in connection with nature and in compliance with their "customs" or cultural norms, they acquire a sacred meaning. Imbalance in any of these aspects can be considered a manifestation of spiritual disconnection, so restoring health also implies a reconciliation with the gods, ancestors, and the natural environment. Thus, spirituality permeates and gives meaning to each dimension of well-being.

In this sense, spirituality plays a fundamental role in maintaining their well-being, as they consider it contributes to harmonizing their lives and strengthening their health, according to the testimony of some participants:

"Spiritual health, that you are connected, that you are conscious... that if we can contribute something to the cosmology, we are obliged to perhaps fulfill what Mother Earth asks for, the solar system, and respect each other, especially nature and water. When you fulfill that, spiritually, with yourself, you are fine..." (A11).

"...coexisting, coexisting with nature, coexisting with nature to breathe good air, to maintain your spiritual balance, knowing how to coexist to live and consume good food... rescuing the culture, fulfilling your culture..." (A4).

While the traditions of the Wixárika people are fundamental for preserving internal and external harmony, as well as preserving their community identity, some participants noted that certain practices may pose risks to physical health. For example, participation in rituals requiring long walks, prolonged fasting, or exposure to the sun for several hours is assumed to be potentially harmful, particularly for vulnerable individuals such as children, pregnant women, or the elderly, who may be at greater risk of dehydration or physical exhaustion. One participant comments: "...through much sacrifice, they overexert themselves, fast too much, and there are people who are strong, but in the end, illness comes to them..." (A3).

Wixárika culture and traditions also reflect tensions that can lead to unhealthy habits. One such situation is the progressive abandonment of Indigenous culture, especially among young people, who, by adopting Western lifestyles, may be exposed to practices that put their health at risk, such as the consumption of alcohol, tobacco, or other substances. A participant explains:

"Problems I see among young people are abandoning the culture and converting or adapting to another culture, like being part of a gang or neighborhoods, learning to smoke, becoming an addict, that is alcoholism, drug addiction. That is the most important thing, the main thing I have seen in the youth of Guadalajara" (A5).

Mental-emotional aspects

When exploring participants' perceptions, it was identified that mental-emotional aspects and the non-use of prohibited substances are also part of their understanding of well-being, especially in relation to tranquility, joy, and good social relationships, as some participants express: "Ah, well, being well physically, emotionally. Or mentally" (C17); "Well, health is feeling good in our physical and mental" (C28).

In this line of thought, one participant expressed that healthcare begins with recognizing one's own value and individual responsibility for the body. He stated:

"Well, first, we have to start loving ourselves because I think we ourselves don't, don't care about our health. And there we go without taking care of ourselves, or sometimes, even if we feel bad, we don't seek care" (C28).

This statement highlights that health, for some, depends not only on personal habits but also on emotional factors, such as self-worth.

Only one participant considered that avoiding drug and alcohol use helps preserve their health, as these substances alter the harmony of the body and mind and their relationship with their community: "...Well, because I don't use drugs, I'm not out there with drunks fighting, I drink a little but I'm not like the others fighting or doing drugs" (A15).

Economic-occupational aspects

In the case of Indigenous people like the Wixárika, economic conditions largely determine the availability of nutritious food, access to health services, housing quality, and possibilities for rest or adequate physical activity. In this sense, only some participants consider the economy as a key component directly impacting the preservation of physical health and general well-being, as one mentions: "...For me, it's being well, with your family, both economically and socially... that you lack nothing..." (A2). For the Wixárika communities that migrated to the city, the economic aspect is very important; another participant comments:

"...Well, not stopping work. Here in the city, you have to be on top of things because if you don't work, you don't bring in money. Here, buses and even going to the bathroom cost you. Here in the city, you have to be working. Because in the communities, in the sierra, at least you have your corn, there you don't worry, you go out to work at different jobs, you irrigate, you find your family, and they eat there..." (A2).

Thus, the physical, spiritual, mental-emotional, and economic-occupational aspects are fundamental for participants to preserve bodily balance and the integral well-being of the participants, as they consider they favor the preservation of their health in harmony with their environment.

The participants in this study do not conceive health in a fragmented way but as the result of a dynamic balance between body, spirit, community, and nature. Attention to diet, hygiene, rest, and physical activity, as well as connection with their gods, the practice of traditional rituals, and the fulfillment of community obligations, are part of a logic that sustains their well-being. Furthermore, aspects such as economic stability and access to basic healthcare resources are also valued as determinants that can strengthen or weaken health. Together, these aspects are articulated in a holistic understanding of life care, where each component plays an essential role in maintaining individual and collective harmony.

Care practices

For the participants, seeking care for any ailment is essential to preserve their well-being and balance. When someone falls ill, they usually first resort to home remedies transmitted from generation to generation, which are part of their ancestral knowledge about nature and its healing properties, as one participant mentions:

"...my son got heatstroke and had vomiting and fever, and my mom did a limpia (spiritual cleansing) on him, I gave him some cumin tea, gave him a natural pill, ground it up, and he took it, and he was like that for two or three days\... with what we have here in herbs, you boil it and see the result, and if not, we take him to the doctor..." (A3).

If the discomfort persists after home remedies, they go to the Mara'akame, the spiritual and healing figure of the community, who, through rituals, prayers, and the use of medicinal plants, seeks to restore harmony between body and spirit. The Mara'akame can offer different treatments for health problems; for example, they may prescribe teas or some home remedy, but they may also offer a limpia with bird feathers or some herb or plant. In some cases, if the Mara'akame considers it very serious, they may request a more elaborate healing ritual. A participant explains this:

"I had an illness which was anemia, the deer's punishment... they took me to a Mara'akame... I was just a skeleton, I remember, I couldn't get up... they made an arrow, because they cure differently, if it's going to be anemia, they cure very differently... he cleansed me, pulled out deer hair from me, if they're a good Mara'akame, they pull out stones, they pull out hair, they pull out corn, and at that moment, you feel like all the bad energy is being pulled out of you..." (A5).

It is important to highlight that some of the illnesses suffered by the Wixaritari are considered punishment for not fulfilling their customs; in these cases, only the Mara'akame can help them improve. One participant comments: "Well, there are illnesses that do come from customs, because one doesn't fulfill what one has to do, and that's when they get sick and go to a Mara'akame" (A9).

The exclusive use of traditional medicine in situations requiring specialized medical attention can delay the diagnosis and timely treatment of serious illnesses. These situations do not diminish the value of ancestral knowledge but highlight the need to promote intercultural dialogue that allows combining traditional knowledge with modern care strategies without delegitimizing the Wixárika people's own beliefs.

Regarding this point, some participants recognize the importance of Western medicine, so if necessary, they also go to the doctor, especially when the illness requires more specialized attention:

"Well, if they get sick, like with the flu, they are given a tea, and then, if they don't improve, they are taken to a Mara'akame, if the Mara'akame is there, it's fine; if not, they look for them. If the Mara'akame doesn't cure them, then they go to the health center..." (A5).

Healthcare practices among the participants reflect a combination of traditional and modern knowledge, adapted according to their context of residence. In communities living in their mountain villages, the use of traditional medicine predominates, where the Mara'akame holds a central place as a spiritual guide and healer, using rituals, sacred songs, medicinal plants, and offerings to the gods. These practices not only seek to cure the body but also to restore spiritual balance. In contrast, those who migrate to urban environments tend to face greater difficulties accessing these ritual elements; therefore, although some continue to resort to healers or home practices, they are also forced to use institutional health services. However, these services often do not consider their cultural values or worldview, which can generate tensions, distrust, or detachment.

Language also represents a significant barrier for Wixárika people accessing medical services in health institutions. This communication limitation hinders mutual understanding between patients and medical staff, affecting both diagnosis and treatment [17]. Added to this is the frequent lack of trained personnel and scarcity of medications in many health units, further worsening the possibility of receiving timely, adequate, and culturally appropriate care.

## Discussion

The study of the worldviews and health practices of Indigenous people is essential for understanding and improving healthcare in intercultural contexts. The Wixárika people, located primarily in western Mexico, possess a rich and complex cosmology that profoundly shapes their understanding of health, illness, and healing practices [18]. Unlike the Western biomedical model, which often focuses on the body as a physical and biological entity, the Wixárika conceive health as a state of holistic balance. This balance encompasses the relationship among the physical, spiritual, mental-emotional, and economic-occupational dimensions. When these dimensions are in harmony, individuals are able to carry out their daily activities, such as household tasks, work, rest, and eating, which are regarded as indicators of health and well-being.

Several studies [1,10,13] support this perspective by emphasizing that Indigenous health models are based on systems of ancestral knowledge and practices that seek harmony between human beings, nature, the spiritual world, and the community. Consistent with our findings, these studies highlight that health is a holistic process interconnected with sociocultural, religious, and environmental dimensions; consequently, illness is perceived as a state of imbalance.

From the Wixárika worldview, physical and spiritual health are not separate entities but inseparable dimensions of life. The body is regarded as the dwelling place of the spirit, and caring for it is understood as an act of respect and reverence. In this sense, everyday practices such as mindful eating, adequate rest, and physical activity transcend self-care habits, as they reflect the value attributed to one's inner being. Mental and emotional aspects are also essential. For the Wixaritari, emotional balance is maintained through community cohesion and harmony with the surrounding environment. Their relationship with the natural world is fundamental: Mother Earth, the sun, and water are part of a sacred bond that sustains life. Through their customs and ancestral knowledge, transmitted from generation to generation, they preserve harmony with nature and find in it the spiritual and communal balance that characterizes them [3,15].

This understanding is not unique to the Wixaritari. Other Indigenous people share similar perspectives, although each has its own distinctive features. Among the Emberá people of Colombia, balance is maintained through a spiritual relationship with Mother Earth; among the Zenú people of Colombia, through a spirituality that permeates daily life and cultural practices; and among the Mapuche people of Chile, health-küme mongen and küme felen-is understood as harmony between nature and the community, encompassing both healthy growth and social values [6]. Recent studies of Indigenous people, including the Mapuche in Chile [11] and the Yánesha in Peru [12], further confirm that health integrates physical, communal, and spiritual dimensions: physical strength is linked to productive work, relational well-being is associated with harmony with nature, and spirituality provides meaning to community life.

For the Wixárika participants interviewed in this study, economic and occupational factors play a central role in maintaining health and well-being. The ability to work and provide for one's family is understood not only as a condition for survival but also as an indicator of physical and mental balance. Family and community economies determine access to food, medicines, and participation in rituals, all of which reinforce collective well-being.

For them, addressing any illness is essential to preserving individual balance. The first step on the path to healing is the use of home remedies passed down through generations [3]. This ancestral knowledge of the healing properties of nature is an integral part of their cultural identity and constitutes the first line of defense against illness. However, their healthcare practices are not limited to traditional approaches; rather, they reflect a combination of ancestral and modern knowledge. Depending on the severity of the illness or the lack of response to home remedies or ancestral healing rituals performed by shamans, individuals may choose to seek assistance from a Western-trained physician. This adaptability demonstrates a pragmatic and flexible healthcare strategy that combines the richness of traditional knowledge with the accessibility of formal medicine, creating a hybrid healthcare system that responds to the needs of their sociocultural context.

Overall, the findings of this study reaffirm that health among Indigenous people should be understood as a complex phenomenon that integrates physical, spiritual, communal, and socioeconomic dimensions. In the case of the Wixárika people, highlighting the role of productive work and the family economy provides an important contribution to the academic debate: well-being cannot be understood in isolation but rather as part of a broader cultural and structural framework, in which interdependence with nature, the community, and the deities defines the very essence of balance and health.

Study limitations

Despite its contributions, this study has several limitations, which should be acknowledged. First, as with all qualitative research, the findings cannot be generalized to the entire Wixárika population, as they reflect the perspectives of 127 adults from selected communities in two specific contexts. Second, although access to the community was facilitated through formal negotiation with traditional authorities, who issued an open invitation to community members, participation was largely determined by who was present at home during the research visits. This may have introduced a selection bias toward women, who constituted 68.5% of the sample, as they were more likely to be at home at the time of the visits. While migration status was identified among participants, it was not used as a selection criterion, which may limit the ability to draw systematic comparisons between migrant and non-migrant subgroups. Additionally, all participants shared a similar socioeconomic background, which not only reduces variability but also limits the exploration of how socioeconomic differences might influence health conceptions. Third, the presence of interviewers and the sensitive nature of some topics, including traditional practices, substance use, spirituality, and healthcare-seeking behavior, may have introduced social desirability bias, with participants potentially presenting responses they perceived as expected or acceptable. Fourth, linguistic and cultural barriers, despite mitigation through bilingual community assistants, may have affected the depth or accuracy of some responses.

Future research should therefore expand the sample to include a larger number of community members and additional age groups, such as children and adolescents; further explore the interactions between traditional Wixárika medicine and the Western healthcare system; and examine the impact of socioeconomic changes, such as migration and globalization, on Indigenous conceptions of health.

## Conclusions

This research focused on exploring the concept of health among the Wixárika people in Jalisco, analyzing its cultural, spiritual, and community dimensions. The study allowed us to understand that for this community, well-being is a state of integral balance that transcends the simple absence of disease. By delving into how they perceive their well-being, we identified certain elements, such as harmony with nature and their gods and community cohesion, as determinants of their physical and emotional balance as self-care practices.

Respecting and integrating Indigenous knowledge into formal health systems may represent a promising pathway toward joint actions aimed at improving the quality of life of the Wixárika people, though this hypothesis warrants evaluation through future interventional research. This collaboration must go beyond mere consultation; it must be a horizontal dialogue that values and recognizes traditional medicine as a valid and complementary knowledge system.
